# Supplementary material for: Predicting visual function by interpreting a neuronal wiring diagram
Source: Nature. 2024 Oct 2;634(8032):113–23. doi: 10.1038/s41586-024-07953-5 (PMC11446822; doi:10.1038/s41586-024-07953-5)
Supplement: Supplementary file 6 — CRF and ERF predictions for individual TmY4 and TmY9 cells. Analogous to Supplementary Data 3, but for TmY target types. Shown are the top four monosynaptic pathways, the strongest pathway passing through each of the top ten intermediary types (ranking from Extended Data Fig. 7), and the trisynaptic pathway Tm1–TmY–Dm3–TmY (see the section entitled Prediction of spatial normalization). [file 41586_2024_7953_MOESM6_ESM.zip › DataS4/TmY9q/720575940624482707.pdf]

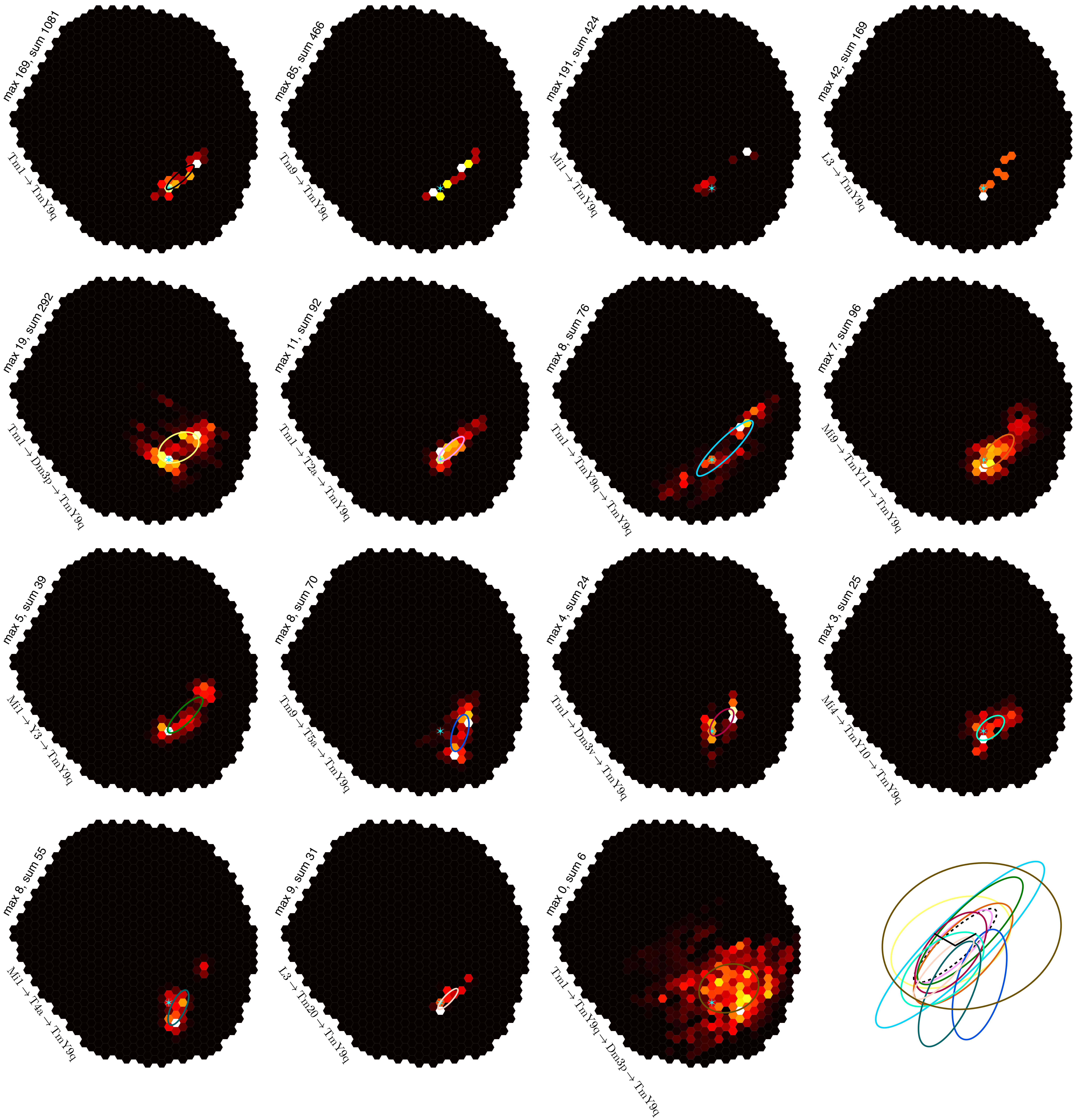
max 169, sum 1081
Tm1 → TmY9q
max 85, sum 466
Tm9 → TmY9q
max 191, sum 424
Mi1 → TmY9q
max 42, sum 169
L3 → TmY9q
max 19, sum 292
Tm1 → Dm3p → TmY9q
max 11, sum 92
Tm1 → T2a → TmY9q
max 8, sum 76
Tm1 → TmY9q → TmY9q
max 7, sum 96
Mi9 → TmY11 → TmY9q
max 5, sum 39
Mi1 → Y3 → TmY9q
max 8, sum 70
Tm9 → T5a → TmY9q
max 4, sum 24
Tm1 → Dm3v → TmY9q
max 3, sum 25
Mi4 → TmY10 → TmY9q
max 8, sum 55
Mi1 → T4a → TmY9q
max 9, sum 31
L3 → Tm20 → TmY9q
max 0, sum 6
Tm1 → TmY9q → Dm3p → TmY9q
